# Realist review of policy intervention studies aimed at reducing exposures to environmental hazards in the United States

**DOI:** 10.1186/s12889-016-3461-7

**Published:** 2016-08-18

**Authors:** Dorie E. Apollonio, Nicole Wolfe, Lisa A. Bero

**Affiliations:** 1 Department of Clinical Pharmacy, University of California, 3333 California Street Suite 420, San Francisco, CA 94143 - 0613 USA; 2 Department of Social and Behavioral Sciences, University of California, San Francisco, USA; 3 Department of Pharmacy; Charles Perkins Centre, University of Sydney, Sydney, Australia

**Keywords:** Pollution, Environmental toxic substances, Systematic review, Health policy

## Abstract

**Background:**

Exposure to pollution is a significant risk to human health. However few studies have attempted to identify the types of policy interventions that can reduce the health risks of pollution exposure in the United States. The study objective was to conduct a realist review of policy interventions conducted or aimed at reducing chemical exposures in humans or the environment where exposure was measured.

**Methods:**

A systematic literature search identified published articles that assessed policy interventions using exposure data. Two coders independently extracted data from the studies, assessing methods, context, details of interventions, outcomes, and risks of bias. Data were analyzed iteratively and manually to identify the most effective and transferrable types of interventions. The reasons for variability in the success of different interventions were explored.

**Results:**

The review found that regulatory interventions that eliminate point sources of pollution appeared to reduce exposure to environmental hazards. Regular monitoring to provide environmental and human exposure data helped assess compliance with the regulatory standards. Educational and economic interventions were less successful.

**Conclusions:**

Although some types of regulatory interventions appear to reduce exposures, our findings are limited by the nature of existing interventions, the weaknesses of the study designs used in the literature, and the lack of details on implementation. Information on contextual factors that influence implementation would assist with future reviews and could help identify effective interventions.

**Electronic supplementary material:**

The online version of this article (doi:10.1186/s12889-016-3461-7) contains supplementary material, which is available to authorized users.

## Background

Exposure to pollution is a significant risk to human health. However few studies have attempted to identify the types of policy interventions that reduce the health risks of pollution exposure. The United States began regulating pollution in the 1960s with the passage of the Clean Air Act. Since then, multiple modifications to that law and others have sought to reduce exposures to environmental hazards that are known to compromise human health. The World Health Organization estimates that over seven million premature deaths annually are caused by air pollution alone. However establishing policies to reduce exposure to environmental hazards requires an evidence base that can identify which kinds of interventions actually reduce pollution and improve human health.

This study provides a realist review of evidence on interventions intended to reduce exposures to environmental hazards in the United States. Realist reviews focus on explanation in addition to outcomes, with the goal of identifying the factors that make particular policies effective. This kind of review is particularly useful in assessing policies and determining whether they can be transferred to other areas. Policymakers express increasing interest in making evidence-informed policy [1–5], but report that they rarely use scientific evidence in making decisions [6–8]. These reports are consistent with research in political science, which reveals that policymakers rely on personal communication rather than written reports and demand customized information [2, 4, 9, 10]. Although policymakers use systematic reviews when the questions considered are relevant, when evaluating policy options they must also assess implementation, which may require a more detailed analysis such as that provided by a realist review.

The goal of this review is to identify the types of interventions that are associated with measureable declines in exposures to environmental toxins. This paper presents the findings and interprets the results of a realist review of 25 studies that assessed policy interventions aimed at reducing exposures to environmental hazards. It summarizes evidence drawn from multiple studies evaluating policy interventions, all of which provide outcome data that specifically measures chemical exposure levels in humans and the environment, in order to determine which US environmental health policies have reduced or prevented exposure to environmental hazards.

## Methods

### Search strategy

This review focused on studies that measured changes in exposure to environmental hazards in either people or the environment. The types of identified interventions were organized according to the following taxonomy [1]: Regulatory (bans, cap-and-trade limits, mandatory reporting) [2]; Economic (taxes, penalties, incentives); and [3] Educational (media campaigns, labeling). Study designs included controlled trials, cohort, interrupted time series, time-series cross-sectional and cross-sectional studies [11].

The assessment of evidence began with a systematic review of the literature. Sources of articles for inclusion in the review included PubMed, EMBASE, Toxline, PAIS International and a Web of Science cited reference search. The literature searches used topic-related keywords and free text terms relating to environmental hazards. Additional file 1 provides details of the full search strategy for each database. Searches were conducted May 12–21, 2014.

The studies included in this review met the following inclusion criteria:

- Published peer-reviewed papers or government reports written in English from 1966 to 2014
- Conducted in the United States as required by the funder Assessed a policy intervention classified as regulatory, economic, or educational
- Measured chemical exposure levels in the environment (e.g., air, water or soil samples) or in humans (e.g., tissue samples), or measured health outcomes directly attributable to chemical exposures (e.g., carbon monoxide poisoning)
- Intervention intended to address an environmental hazard (e.g., lead exposure, auto exhaust or other airborne pollutants, water pollution, hazardous waste).

The search strategy applied the following exclusion criteria:

- Non-English publications
- Multiple publications from a single study (in this case, the most comprehensive study was included)
- Interventions measuring exposure in animals only
- Interventions measuring prenatal exposure
- Tobacco exposure
- Water contamination from pharmaceutical or illegal drug waste
- Radiation exposure
- Workplace asbestos, radon, or beryllium exposure
- Occupational pesticide exposure
- Biological allergen exposure (e.g., dust, mildew, mold).

Upon reading articles and beginning data extraction, some articles were excluded upon finding that they did not meet the inclusion criteria. Most commonly this meant a study did not report on exposure to environmental toxins. For example, one study discussing nonpoint source pollution in waterways only presented findings on soil loss, while another study reviewed policies aimed at reducing exposures but did not report the exposure data used in developing those policies. Other studies were excluded on the grounds that although they reported on exposure data, they did not discuss a policy intervention. For example, one paper presented findings for a calibration study of identical samples tested by different laboratories. This paper did not involve research on human subjects. As there were no participants, neither ethics committee approval nor informed consent were sought or obtained.

### Data analysis

#### Analytical framework

This review relies on a realist perspective for data analysis [12–14]. Realist reviews seek to answer the question: “What is it about this policy that works, for whom and in what circumstances?” [14] Realist reviews focus on explanation in addition to description, with the goal of identifying the factors that make particular policies effective. From this perspective, policy interventions occur within different environments, and these interactions create reactions (mechanisms) that cause particular outcomes. Such interactions are referred to as “context-mechanism-outcome configurations” [12].

Interpreting context-mechanism-outcome configurations implies an understanding of the nature of each parameter. The **context** is the circumstances or environment in which policy interventions are implemented. These may include organizational, socioeconomic, cultural, and political conditions, the perceptions of stakeholders, and the process of implementation. The **mechanism** is the reaction to the intervention, which may be created intentionally or unintentionally depending on the context. These interactions may or may not lead to the intended **outcome**.

**Fig. 1:**
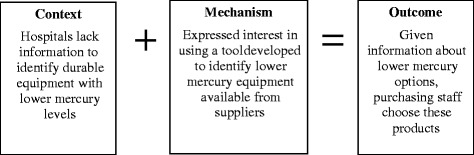
Sample context-mechanism-outcome configuration for an economic study of environmental purchasing [15]

Assessing evidence using realistic review addresses context, mechanism, and outcomes by explicitly stating the assumptions of policymakers regarding the expected outcomes and how they will be achieved, and researching the implemented intervention to determine whether these assumptions were borne out. An example of a context-mechanism-outcome configuration for one of the studies included in this review is provided in Fig. 1. This study, which assessed an economic intervention, sought to determine whether an environmental purchasing program could reduce mercury waste at health care facilities in the Great Lakes region [15]. The **context** was an environment in which health care facilities lacked information about the mercury levels in different potential products available for purchase. The researchers created an intervention, the Health Care Environmental Purchasing Tool, which asked the facilities’ suppliers to provide information about mercury levels in products. The researchers provided an incentive payment for health care facilities to pilot the tool in their purchasing departments. The **mechanism**, or the reaction triggered by the intervention, was positive interest in using the tool, particularly given that it was developed in concert with purchasing officials and in light of institutional sustainability goals and the incentive payments. Using the tool led purchasers to the **outcome**, which was change in purchasing patterns that resulted in both one-time and annual reductions in mercury waste.

The realist review approach is designed to address complex policy questions, and synthesize evidence from multiple settings and interventions. Research based on a realist perspective can use qualitative, quantitative, or mixed methods of data collection and analysis. This review, which relies on measures of chemical exposure, considers quantitative results. A realist review systematically identifies and assesses the results of existing studies. It focuses identifying the mechanisms that resulted from the interaction between the intervention studied and the context, which allows what is referred to as a generative approach to causality. This approach allows specific consideration of the possibility that particular policy interventions could be transferred to other settings [13].

#### Analysis

In order to identify key elements critical to the success or failure of policy interventions, information gathered from studies included specifics on the nature of interventions, the context, and their implementation, or success of the intervention, as assessed by measurements of chemical exposures.

One author (NW) extracted background data on the included studies (author, title, journal) using a standardized form prior to coding for content. Two authors (DA, NW) independently extracted data on content and risk of bias for all included studies, double-coding 20 % of the included studies to assure consistency and objectivity. Double-coded articles were discussed until findings reached consensus. All information was drawn from studies using a tool developed and modified by two of the authors (DA, LB). Information drawn for analysis included [1]: methods, including the type of intervention, study site, study design, aims, inclusion and exclusion criteria, outcome measures, outcome verification (if any), and methods of analysis [2]; context, including the targeted population and sample size [3]; intervention and assessment details, including exposure, duration of the intervention, the time between intervention and assessment, comparisons, and effect modifiers [4]; outcomes, detailing the results of the intervention [5]; risks of bias, including selection bias, concealment of allocation, incomplete outcome data, and any other potential threats to validity.

Articles were read as transcripts of the research conducted and the coders extracted information regarding contextual factors, the reactions triggered by the intervention, and statements of how the intervention was intended to work. Only the reported findings were listed in the data extraction tool, rather than coder interpretations. A sample of the data extracted using the tool is provided in Appendix 1. This study measured air quality before and after a regulatory intervention [16].

The data analysis was performed iteratively and manually. Preliminary results were discussed between the two researchers extracting data (DA, NW), the report was drafted by one of the researchers (DA), and was reviewed and revised by the principal researcher (LB).

## Results

### Summary of evidence

**Fig. 2:**
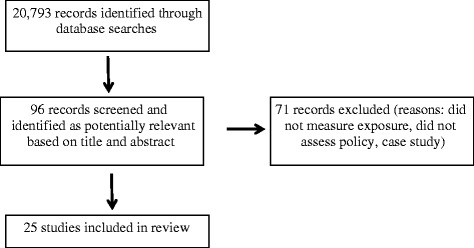
Study flow diagram

This section provides details about the types of studies, the level of evaluation of the intervention, and the data-collection methods. The final search identified 25 studies that met the inclusion criteria. Table 1 lists the study designs; although one study relied on a design generally recognized to be high-quality, a randomized controlled trial, most rely on observational data, including cross-sectional and time-series cross-sectional data that use before and after measurements as controls or use no controls. The hierarchy of evidence for assessing interventions assesses the study designs included in this review as fair to excellent, with none of the included studies relying on poor-quality study designs (e.g., expert opinion) [17]. The 21 included studies were classified consistent with the study goal, which was to compare the effectiveness of different types of interventions: 21 evaluated a regulatory intervention, 2 evaluated an educational intervention, and 2 evaluated an economic intervention. An additional 71 studies were identified and excluded, because the study did not provide measurable outcomes, the outcome measured was not a chemical exposure, or the study did not analyze a policy intervention. Figure 2 provides a study flow diagram.

**Table 1:** Study characteristics by type of intervention

Intervention type	Exposure type	Study	Outcome measures	Measurement type	Study design	Study site
Regulatory (*n* = 21)	airborne pollutants	Bahadur 2010	black carbon	environmental	interrupted time series	California
		Gego 2007	ozone	environmental	interrupted time series	Eastern US
		Aleksic 2013	ozone	environmental	interrupted time series	New York
		Cheung 2005	particulate matter	environmental	interrupted time series	Los Angeles Basin, CA
		Thomas 2013	sulfur	environmental	cross-sectional	Potomac River, WV
		Dallman 2011	multiple pollutants	environmental	interrupted time series	Oakland, CA
		Bishop 2013	multiple pollutants	environmental	interrupted time series	South Coast Air Basin, CA
		Davis 2012	multiple pollutants	environmental	time series cross-sectional	California
		Pokharel 2013	multiple pollutants	environmental	time series cross-sectional	multi-city data set (USA)
		Lin 2013	respiratory diagnosis	human	time series cross-sectional	New York
		Mott 2002	carbon monoxide related deaths	human	interrupted time series	national data set (USA)
	lead paint	Brown 2001	blood levels & hard surfaces	human, environmental	retrospective cohort	2 northeastern states (USA)
		Galke 2001	blood levels & hard surfaces	human, environmental	interrupted time series	multi-city data set (USA)
		Rich 2002	hard surfaces tested	environmental	randomized controlled trial	New Jersey
		Breysse 2007	hard surfaces tested	environmental	interrupted time series	Baltimore, MD
	water pollution	Lakind 2010	trihalomethane (THM) levels	human	time series cross-sectional	national data set (USA)
		Dorsey 2010	fecal indicator bacteria	environmental	interrupted time series	California beaches
		Hundal 2014	trace metal concentrations	environmental	interrupted time series	multi-city data set (USA)
		Daberkow 2001	nitrate-nitrogen levels	environmental	time series cross-sectional	Central Platte Valley, NE
		Kauffman 2011	multiple pollutants	environmental	interrupted time series	Delaware Basin, DE
	pesticides	Clune 2012	pesticide levels	human	interrupted time series	national data set (USA)
Educational (*n* = 2)	lead paint	Aschengrau 1998	blood levels & hard surfaces	human, environmental	non-randomized controlled trial	Boston, MA
	water pollution	Postma 2011	well water contaminants	environmental	cross-sectional study	Montana & Washington state
Economic (*n* = 2)	airborne pollutants	Lu 2012	sulfur dioxide emissions	environmental	time series cross-sectional	national data set (USA)
	hazardous waste	Eagan 2002	mercury levels	environmental	interrupted time series	Great Lakes region

#### Description of interventions

The studies included in this review covered three primary types of policy interventions. The summary of findings in Table 1 provides an overview of the taxonomy of interventions, the number of studies that covered each type of intervention, the nature of the outcomes included in the review, and whether the study measured exposures in humans or the environment. We categorized the studies by the type of intervention (regulatory, economic, or educational) in advance to reflect the different types of policy approaches taken to reducing environmental exposures.

The types of policy interventions designed to reduce exposures to environmental toxins fell into three categories. The first type, regulatory interventions, includes laws designed to eliminate exposures through bans or requirements for modifications on equipment producing toxins. The second type, economic intervention, includes changes that provide a financial incentive in service of a reduced environmental exposure. The third type, educational interventions, includes policies designed to inform at-risk populations how to reduce their own exposure.

#### Research design

The studies included in this review did not always describe the nature of the interventions assessed, making it different to compare studies. Moreover, only a minority of studies provided baseline data to compare with the post-implementation measures intended to identify the effects of interventions. Table 1 shows the different types of research designs that were used. Two studies used controlled trial designs, one randomized, with control and intervention groups to allow comparisons (Rich 2002 randomized, Aschengrau 1998 nonrandomized). One study used a retrospective cohort design (Brown 2001), which included both a control group and baseline data. Of the 25 total studies, the majority, 14 studies relied on an interrupted time-series design, where measures were taken before and after an intervention was implemented (see Table 1). These studies all lacked control groups. The remaining studies relied on cross-sectional or time-series cross-sectional data, where measures were taken at a single point in time in a single sample (Thomas 2013, Postma 2011) or at multiple points in time from new samples (6 studies, see Table 1). All of the included studies provided at least one quantitative measure of outcomes, in the form of data detailing exposure levels or their proxies.

#### Implicit and explicit assumptions

All interventions had a logic that underlay the design of the policy. The restriction of this review to studies that explicitly measured exposure data meant that all of the studies included in the analysis stated the intended outcome of the intervention. The reliance on existing datasets did not always make it possible rule out alternative explanations. For example, one study of the impact of the EPA (Environmental Protection Agency) NOx (mono-nitrogen oxides) Budget Trading Program reviewed New York state hospital discharge records for respiratory diagnoses after the implementation of the program (Lin 2013), but could not control for the simultaneous implementation of the state’s clean indoor air law, although implementation of this law would also be expected to affect levels of respiratory disease [18]. In addition, the use of existing datasets to measure exposures meant that the policy intervention may have had additional effects that could not be measured with the data that were available to researchers.

#### Risk of bias

A majority of the included studies (15 of 25) described problems with incomplete outcome data; these issues are described further in results. In all of these cases, the method used to address missing data was exclusion of cases. Selection bias was also noted as a concern; five of the 25 included studies noted that the sample data were non-representative in some way, due to the use of either a convenience sample or a volunteer population. Only one study used a randomized controlled trial design (Rich 2002); the methods used for allocation and concealment (blinding) were not described. The risks of bias identified here may underestimate the actual risks of bias, as these issues were typically not discussed in the included studies.

Although measures of exposure identified by the inclusion criteria could be assessed in either the environment or in humans, the overwhelming majority of studies (18 of 25) reported only environmental measures.

The types of environmental exposures studied fell into five categories: (a) airborne pollutants, (b) hazardous waste, (c) lead paint, (d) pesticides, and (e) water pollution. A total of 12 regulatory and economic studies reviewed policy interventions and outcomes aimed at reducing airborne pollutants (see Table 1). All of these studies relied on environmental measures. Only one study of an economic intervention sought to assess reductions in hazardous waste; relying on environmental measures. Similarly, a single study addressed pesticide exposure using measures in humans. An additional five studies considered regulatory and educational interventions directed at reducing exposure to lead paint. Of these, two studies relied on the environmental measure of hard surfaces testing, and three studies used measures of both environmental and human exposures. A total of six studies assessed regulatory and educational interventions directed at water pollution; of these, five relied on measures of environmental exposures and one on human exposure data.

The next sections review the data analysis in each category of intervention, describing first the characteristics of the interventions and their outcomes, then the context and mechanisms. The extraction tool and sample article in Appendix 1 details the kinds of data drawn from each article.

### Findings and analysis by type of intervention

#### Regulatory interventions

In total there were 21 regulatory interventions covering four types of exposures: airborne pollutants, lead paint, water pollution, and pesticides (see Table 1).

##### Airborne pollutants

*Study characteristics:* Of the 21 regulatory intervention studies, 11 focused on airborne pollutants. These included regulation of fuel additives, increasingly stringent vehicle emissions standards, explicit requirements to retrofit or replace existing vehicles, various air quality regulations encompassing a range of required reductions in pollutant levels, and nitrogen oxide emissions reduction standards. Nine of the included studies relied on environmental measures, and two used measures of effects on humans. Seven of the 11 studies reviewed data from before and after the implementation of the intervention(s) [16, 19–24] and the remainder included only post-implementation measures [18, 25–27].

*Outcomes:* Regulations of vehicle fuels and emissions standards were the most commonly studied form of policy intervention addressing airborne pollutants, making up over half of the 11 included articles. Bahadur 2010 studied increasing restrictions on diesel fuel in California using data collected by the California Air Resource Board to show a statewide decline in black carbon levels [19]. Davis 2012 focused on reductions over time in allowed vehicle carbon monoxide levels to show that multiple pollutants measured by the California Air Resource Board declined with increasingly stringent regulation, even when controlling for reduced driving during recessions [25]. Two studies specifically considered changes in vehicle emissions controls standards [21, 26]. Both collected data on particulate matter and other pollutants using road sensors, one in three different cities (Pokharel 2013) and one at multiple sites around greater Los Angeles (Bishop 2013). Both studies showed decreasing levels of pollutants over time with the implementation of increasingly stringent air quality standards. Two other studies reviewed requirements to retrofit or replace vehicles that had been identified as key sources of pollutant exposure. Mott 2002 focused on the requirement to add catalytic converters to U.S. vehicles and showed steady reductions on carbon monoxide-related deaths using national death certificate data over more than 30 years [24]. Dallman 2011 assessed air quality by measuring multiple pollutants released into the air above diesel trucks entering the Port of Oakland before and after the implementation of a requirement to retrofit truck filters or replace older trucks with new vehicles. It found a significant decrease in levels of measured pollutants [16].

The remaining studies reviewed broader air quality interventions. Three studies specifically considered policies that required reductions in nitrogen oxides emissions. Gego 2007 used data drawn from federal monitoring sites in the eastern United States and found reductions in ozone levels despite incomplete implementation of the law [22]. Aleksic 2013 specifically assessed ozone levels in New York before and after implementation of the standards using data from federal monitoring sites and also found significant declines [23]. Lin 2013 specifically assessed a nitrogen oxides emissions reduction Budget Trading Program, which was correlated with a decline in hospitalizations based on a respiratory diagnosis after implementation [18]. Cheung 2005 sought to assess the effects of a range of air quality regulations implemented over time. This California-specific study in the Los Angeles Basin found reductions in particulate matter over time [20]. Thomas 2013 measured sulfur levels in juniper trees after the implementation of the Clean Air Act, and found both reduced emissions and a recovery in the health of the trees [27].

*Contextual factors and mechanisms:* The theory behind regulatory interventions is that removing products that expose individuals and the environment to toxins associated with adverse health outcomes will reduce or eliminate that exposure. Context and mechanism were not systematically discussed in these studies. When they were discussed, most attention was paid to the problems with describing the context in which interventions were made.

Regarding context, all of the interventions took place in the United States, and were measuring outcomes that could be affected both by the specific interventions studied and by additional regulatory changes, which typically occurred at the federal level. Many of the studies did not attempt to describe the effects of specific interventions, assuming instead that the combined effect of additional regulations was causally related to the outcomes of reduced pollutant levels. This assumption was not always plausible. For example, Lin 2013, which focused on hospital admissions for respiratory diseases in the wake of nitrogen oxides regulation, noted that the simultaneous passage of a New York state clean air law targeted at tobacco use was also likely to have affected the outcome measure [18].

The specific mechanisms triggered by the interventions were rarely described. For some of the studies, those in compliance or out of compliance with regulations may not have been aware of the implications of their behavior at all.

*Interpretation:* The studies included rarely provided an assessment of individual interventions, making it difficult to draw definitive conclusions about the effectiveness of particular policies. Although all of the studies of air quality regulations were correlated with reduced levels of airborne pollutants using multiple measures, most of the study designs did not allow an assessment of which interventions led to changes in outcomes. The confounding factors of multiple simultaneous interventions that were not measured were noted in many of the studies.

Nevertheless, some studies specifically assessed changes in measured pollutants before and after the implementation of individual interventions. Studies at the local and regional level that reviewed air quality measures before and after the implementation of specific interventions suggested that two kinds of interventions reduced exposures: (a) those requiring retrofit or replacement of known producers of airborne pollutants (e.g., fuels, filters, vehicles), and (b) interventions that required reductions in the levels of certain pollutants as independently verified by federal monitoring, but that allowed state and local governments to choose the mechanism by which to achieve these reductions.

These studies suggest that the findings of the larger national studies, although they lack controls for effect modifiers, may be valid as well. Interventions that eliminated known sources of airborne pollutants reduced environmental exposures. Where federal monitoring was in place to measure air quality outcomes, interventions that established a maximum standard and allowed states and localities to develop their own plans to meet it also appeared to be successful. All of the regulatory studies of airborne pollutants showed that regulatory interventions at least partially reduced exposures.

##### Lead paint

*Study characteristics:* Four of the 21 studies of regulatory policies addressed lead paint exposure and remediation (see Table 1). In addition, one of the educational studies also addressed lead paint remediation, and is discussed in more detail below. Lead paint abatement efforts have a long history in the United States. Although the primary source of exposure in humans, lead paint, was banned in 1977, older buildings may still contain paint with levels of lead that can cause significant health hazards. Leaving it untouched poses persistent risks because lead paint can flake or dust off over time, and tastes sweet, which leads children to consume it. Brown 2001 and Galke 2001 reviewed a range of housing and remediation policies across multiple sites before and after the implementation. Rich 2002 tested cleaning methods to reduce lead exposure in New Jersey, and Breysse 2007 assessed lead treatment required by law in Baltimore, MD. Rich 2002 and Breysse 2007 used environmental measures exclusively, the testing of hard surfaces [28, 29]. Brown 2001 and Galke 2001 included both the environmental measure and a measure of human exposure using blood samples from children in residence at the affected sites [30, 31]. All studies used research designs that collected data before and after the intervention; Rich 2002 was a randomized controlled trial and the Brown 2001 was a retrospective cohort study.

*Outcomes:* The four studies considered multiple interventions that could reduce lead exposure. Rich 2002 tested cleaning techniques, comparing standard practices to more or less intensive methods. While all of the interventions reviewed were successful in reducing lead dust measured on hard surfaces, the combination of cleaning with TSP (trisodium phosphate) and vacuuming with a HEPA (high-efficiency particulate air) filter was most effective [28]. Brown 2001 compared strict enforcement of lead testing and remediation housing policies in one state to less-strict enforcement in a second state, and determined that the stricter enforcement was associated with lower blood levels of lead as well as higher property values [30]. Galke 2001 used multiple sites to review a range of HUD (US Department of Housing and Urban Development) interventions developed by local governments; these interventions were not described in detail. The study included an assessment component and determined that all measures reduced exposures, and that lower-intensity interventions that did not include abatement could also do so [31]. Breysse 2007 reviewed lead treatments required in the city of Baltimore by law, including an assessment of whether visual inspection was sufficient to identify risk. The study suggested that visual inspection was significantly less effective than testing of hard surfaces, and that although lead dust levels declined after remediation they remained above recommended levels [29].

*Contextual factors and mechanisms:* Context and mechanism were not described in detail in these studies. None of the three studies that discussed local interventions (Brown 2001, Galke 2001, Breysse 2007) specifically detailed the interventions that were used to reduce lead exposure. Although Brown 2001 proposed that stricter enforcement could reduce lead exposure, the authors noted that the direction of causality for the finding of association was unclear. It is possible that areas with higher property values had more resources available for enforcement as well as for remediation of lead risks [30]. The Rich 2002 study of cleaning techniques noted that HEPA-filter vacuums, although they reduced exposure, were both more expensive and more difficult to purchase in poor, urban areas where the risks of lead exposure were higher. As an alternative, the authors noted that more frequent cleaning could compensate to some extent for less effective equipment [28]. The Breysse 2007 study of lead remediation interventions in Baltimore suggested that objectively measuring of outcomes through testing was more effective than visual assessment by inspectors [29]. Consistent with this finding, the Galke 2001 multi-site study of local interventions that showed a range of interventions reduced exposure also included an assessment component [31].

The mechanisms, or reactions triggered by the interventions, were not described in any of the four studies, although the authors of the Rich 2002 study of cleaning methods speculated about possible public responses to a change in recommendations based on their findings. The authors of the Brown 2001 enforcement study advised directing more resources toward stricter enforcement of lead abatement policies, but competing priorities for those resources were not described, as this proposal was hypothetical. The mechanisms in the other two studies could not be described because the interventions themselves were not fully described.

*Interpretation:* The studies of regulatory lead paint abatement interventions suggest that these interventions can reduce exposure. Stricter enforcement and testing were associated with reduced exposure, as well as more intensive remediation efforts. As with regulatory interventions in other areas, monitoring results and requiring removal of known pollutants improved outcomes.

##### Water pollution

*Study characteristics:* Of the 21 regulatory intervention studies, 5 focused on water pollution. These included a grant program to improve water quality at beaches (Dorsey 2010), a voluntary fertilizer management program (Daberkow 2001), a modification of drinking water purification standards (Hundal 2014), and two assessments of multiple interventions intended to improve water quality that were implemented over several years (Kauffman 2011, Lakind 2010). Four of the five studies relied on environmental measures, and one used measures of effects in humans (Lakind 2010.) Dorsey 2010, Kauffman 2011, and Hundal 2014 reviewed data from before and after the implementation of the intervention(s) [32–34], and Daberkow 2001 and Lakind 2010 reviewed only post-implementation measures [35, 36].

*Outcomes:* Regulatory interventions that sought to address water pollution were divided in terms of scope, with two studies (Lakind 2010, Hundal 2014) reviewing national-level outcomes and the rest reviewing regional data. The national studies included one study of environmental exposure in humans. After the implementation of a new drinking water disinfection rule that requiring the reduction of pollutant levels post-treatment, Lakind 2010 used national blood sample data drawn from NHANES (The National Health and Nutrition Examination Survey), found that one of the targeted pollutants decreased in the blood samples but the other targeted pollutants did not [36]. Hundal 2014’s study of the Clean Water Act assessed trace metal levels in sludge after implementation using EPA data and found both that trace metal concentrations declined significantly and that the post-implementation sludge could be classified as “exceptional quality” [34]. Kauffman 2011 assessed multiple interventions, including not only federal water quality standards but the establishment of sewage treatment plants and phosphate detergent bans. Using data collected by USGS monitoring stations, it found that overall water quality along the Delaware River significantly improved, and measured levels of multiple pollutants declined [33].

The other studies specifically considered state-level interventions to reduce water pollution. Dorsey 2010’s study of the California Clean Beach Initiative measured water quality at multiple sites that had successfully bid for grant funding to develop programs to improve local water quality. Fecal indicator bacteria were measured before and after the program implementation and the interventions with the greatest reductions were assessed as evidencing the best intervention practices [32]. Daberkow 2001’s study of Nebraska’s efforts to meet federal drinking water standards through a fertilizer management program showed that providing advice on appropriate usage levels led to a decline in fertilizer use and associated pollutants, but that both remained above recommended levels [35].

*Contextual factors and mechanisms:* As in other areas of exposure, context and mechanisms were not systematically discussed in these studies. Similar to the studies of airborne pollutants, the main consideration was the context in which interventions were made.

The studies of regulatory interventions targeting water pollution were evenly split between assessments of national policies and state policies. The efforts to assess context are hampered by the fact that only one of the studies of national policies, Lakind 2010, could describe the effects of specific interventions. For two studies (Kauffman 2011, Hundal 2014) the period of analysis included implementations of multiple interventions. Studies of state-specific policies (Dorsey 2010, Daberkow 2001) were better able to assess the effects of specific interventions. Studies of both national and state interventions were more successful in measuring outcomes when data that could measure compliance were available or when data collection had been built into the intervention. Lakind 2010’s study of changes in blood pollutant levels after the establishment of new drinking water disinfection standards was sponsored by the industry that developed the new disinfection process; the effect of this potential conflict of interest was not discussed by the authors [36].

The mechanisms triggered by the interventions were not fully described. The studies that included multiple interventions (Kauffman 2011, Hundal 2014) could not disentangle mechanisms, even if there had been an effort to describe them. Studies that use anonymized national data on human exposure such as NHANES, like Lakind 2010, cannot assess the reaction even to specific interventions. Studies of single interventions (Dorsey 2010, Daberkow 2001) may also find that the reactions triggered by the intervention were not those that had been anticipated. Daberkow 2001 relied on providing advice to farmers as a means to reduce fertilizer use, but noted that this advice was undercut by the farmers’ overly optimistic assessments of expected crop yields, which led to continued over-fertilization and as a result, to unexpectedly high levels of water pollution [35]. Studies of interventions that provided a strict standard, measurable outcome data, or a clear enforcement mechanism showed reductions in pollutants, possibly by making the implications of meeting or not meeting the regulatory standard apparent.

*Interpretation:* The five studies on water pollution interventions showed the anticipated results for some interventions. Studies that considered multiple interventions could not determine the effectiveness of any particular policy. Even when a specific policy could be studied, as in the case of a new drinking water disinfection method in Lakind 2010, the study showed mixed results. Moreover, the Lakind 2010 study was funded by the organization that created the disinfection process, which raised issues of potential conflict of interest.

The studies of specific state-level interventions suggested that monitoring of outcomes may identify policies that reduce measured pollutants. Dorsey 2010’s study of the California Clean Beach Initiative monitored five types of interventions proposed by local agencies for funding and was able to determine which intervention was associated with the greatest decrease in fecal indicator bacteria (diversion and a combined approach). Daberkow 2001 found that efforts to decrease fertilizer use in Nebraska by providing advice to farmers, which did not require changes to behavior or link reductions in fertilizer use to incentives or monitoring, were less effective.

##### Pesticides

*Study characteristics:* The final study of a regulatory intervention, Clune 2012, reviewed the outcomes of a reduction in organophosphorus (OP) insecticide use under the Food Quality Protection Act of 1996. It measured outcomes in humans, specifically levels of pesticide concentrations in urine samples before and after the implementation of the intervention that were drawn from a national dataset (NHANES) [37].

*Outcomes:* Clune 2012’s study, which was national in scope, found that measures of pesticide concentrations in urine declined significantly after implementation of the new standard [37].

*Contextual factors and mechanisms:* Clune 2012 noted that use of a national dataset made it difficult to determine the exact cause of changes in human exposure levels. Their analysis did not indicate whether the change in law was the proximate cause of the reduced exposure in humans, as it was possible (although unlikely) that producers and consumers decided independently to stop using all types of pesticides prior to the implementation of the law eliminating sales of OP insecticides. However measures of other environmental exposures in the same population suggested that the use of unrestricted pesticides did not decline, as the human exposure outcomes associated with the use of these chemicals did not decline. The mechanism, or reactions to the intervention, was not assessed; as noted above, this is rarely possible when using an anonymized dataset to measure human exposures.

*Interpretation:* Consistent with findings from regulatory interventions in other areas, banning the sale of a product known to be associated with health risks reduced exposure.

#### Educational interventions

The review included two studies that considered education interventions, which addressed two types of exposures: lead paint (Aschengrau 1998) and water pollution (Postma 2011).

*Study characteristics:* The two educational studies assessed efforts to teach families how to reduce their environmental exposures. Aschengrau 1998 was a controlled trial that sent outreach workers to the intervention group to teach caregivers how to clean housing units where children were exposed to lead paint [38]. Postma 2011, a cross-sectional survey, encouraged families to test well water for contaminants [39]. The Aschengrau 1998 study of lead remediation instruction reviewed measures of environmental exposure (hard surfaces testing) as well as human exposure (blood levels) before and after the intervention. The Postma 2011 study of well water contaminants assessed measures of environmental contamination after the intervention.

*Outcomes:* The Aschengrau 1998 educational intervention that sought to address lead paint exposure was assessed at the local level, in the city of Boston. Outreach workers made a home visit to participants in the intervention groups to educate caregivers how to remove lead dust through cleaning in order to reduce exposure, but after the intervention, researchers found that caregivers did not complete all of the suggested cleaning, particularly in window wells. The intervention was only effective for children that had severely high lead levels, and showed no effect or a negative effect in less severe cases [38]. The Postma 2011 study of well water contaminants after an educational outreach effort in Montana and Washington found that 27 % of households tested positive for at least one contaminant post-intervention, and that 89 % of wells contaminated with coliform in the first test were still contaminated with coliform at retest. Higher socioeconomic status was correlated with higher testing rates before the intervention, and with a lower risk of exposure to pollutants [39].

*Contextual factors and mechanisms:* The theory behind educational interventions is that informing the populations at risk of their exposure and of remediation strategies can change behavior in such a way that exposure levels decrease. The educational intervention studies addressed context more than the studies of regulatory interventions. The targeted populations of these interventions were at higher risk, due in many cases to poverty. Although the need for protection from environmental exposures in these groups is higher, because their living situations are more likely to expose them to pollutants, their ability to remediate these risks is lower due to limited resources.

The mechanism, or the reaction to the intervention, suggests that the value of educational interventions may be limited. Caregivers targeted by the lead paint educational intervention studied by Aschengrau 1998 did not complete the suggested cleaning strategies, leaving children at risk of continued exposure to lead. The studies of lead paint regulatory interventions, discussed above, suggest that these caregivers may not have had the resources available to clean as advised. Similarly, the efforts by Postma 2011 to provide information about well water contaminants did not necessarily lead to remediation, given that nearly 90 % of households with well water contaminated by coliform on the first assessment still had contaminated water on the second assessment. In both cases, there was inadequate response to the intervention, suggesting a broken mechanism. This may have been caused by an incomplete understanding of the context in which interventions were undertaken.

*Interpretation:* Educational interventions did not appear to reduce exposure to environmental hazards. Both efforts to educate individuals exposed to environmental toxins, with the expectation that they could remediate these risks themselves, were unsuccessful. This failure may reflect the fact that individuals at highest risk of exposure are also least likely to have the resources available to pursue remediation.

#### Economic interventions

The review included two studies that considered economic interventions, which addressed two types of exposures: airborne pollution (Lu 2012) and hazardous waste (Eagan 2002).

*Study characteristics:* Lu 2012 considered the effects of changing natural gas prices relative to regulation on national sulfur emissions levels, after passage of the Acid Rain Program and cap-and-trade regulation [40]. Eagan 2002 assessed a purchasing tool that provided information on mercury levels in products for reducing waste mercury production by health care facilities in the Great Lakes region [15]. Both studies reviewed measures of environmental exposure, specifically sulfur emissions tracked by the EPA (Lu 2012), and the extent of mercury waste produced by health care facilities in the study sample (Eagan 2002).

*Outcomes:* The economic intervention study by Lu 2012 assessed the effect of changing natural gas prices relative to national regulation on sulfur emissions and was conducted at the national level. Lu 2012 found that the drop in sulfur emissions was attributable to the regulatory interventions rather than the economic effects of changing natural gas prices [40]. Eagan 2002’s study of hazardous waste focused on developing a tool for health care facilities in the Great Lakes region to voluntarily reduce mercury waste by purchasing products that contained lower levels of mercury. The intervention developed was a purchasing tool sent to suppliers which provided information on the mercury levels in each product, combined with an incentive payment that encouraged participating health care facilities to pilot use of the tool. After the intervention, new purchases reduced mercury waste both in durable equipment (a permanent reduction) and in annual purchases (an annual and ongoing reduction). Five of the nine participating health care facilities stated that they would continue to use the purchasing tool in the future [15].

*Contextual factors and mechanisms:* The theory behind economic interventions is that changing the relative prices of products that produce lower environmental exposures can change behavior. The context for these interventions suggests that this theory may be borne out in some circumstances but not others. In the national study conducted by Lu 2012, an external economic intervention did not change environmental exposure, and given the aggregate nature of the data, it is difficult to interpret the context, which probably varied substantially based on the types of purchasers. The regional study by Eagan 2002 focused on health care facilities that voluntarily enrolled in a purchasing program intended to reduce hazardous waste, and provided a financial incentive for them to learn to change their purchasing behavior. In addition, it targeted a population—purchasing departments—with specific experience in economic interventions.

The mechanism, or reaction to the intervention, reflected the different populations included in the studies. The population that voluntarily enrolled in the Eagan 2002 study was responsive to the intervention, and changed behavior to reflect the goal of reducing waste mercury. Even so, nearly half of the participants stated they would no longer use the intervention after the end of the study and its incentive payments. The population subject to a natural experiment in Lu 2012—changing gas prices—was not responsive to the economic intervention. The contemporaneous regulatory interventions appeared to have more effect in reducing exposures.

*Interpretation:* The studies of economic interventions suggest that they only work in certain contexts. With a population that volunteered to test an intervention in Eagan 2002, engagement with the process was high and the effect was to reduce hazardous waste. Measurement of exposure and of outcomes provided feedback to participants about what strategies would be successful. The economic intervention studied by Lu 2012 that relied on a natural experiment with gas prices was less successful. Although it was impossible to assess the mechanism, it is unlikely that the intervention, in the form of higher prices, was viewed as a means to reduce exposure to environmental toxins. Moreover, any effects of the intervention on exposure levels would not have been evident until well after the fact. Economic interventions appear to be most appropriate when used in populations that are willing to change behavior but lack information. Even in these circumstances, continuing subsidies may be required to maintain full participation.

## Discussion

### Summary

This review suggests the following preliminary conclusions regarding policy interventions to reduce exposure to environmental hazards. First, regulatory interventions that require the elimination of known sources of pollutants appear to be the most successful strategy for reducing exposure to environmental hazards. This finding may reflect the concentration on these strategies in the existing literature assessing policy interventions; however this finding is consistent with the context and mechanisms described in the studies. For example, all of the studies of airborne pollutants, lead paint and pesticides that assessed interventions which reduced or eliminated production of pollutants (e.g., emissions reductions standards, fleet replacements, interventions to remediate lead paint in hours, bans of OP pesticides) showed significantly reduced exposures to environmental hazards. Second, consistent monitoring of environmental and human exposure data is necessary to identify when interventions have been successful. Given an appropriately designed intervention study, regular collection and analysis of monitoring data could also identify which specific interventions reduce pollutants.

Providing advice and relying on voluntary reductions in the production of pollutants was not associated with significant reductions in exposure to environmental hazards. Providing education and training to individuals at risk of exposure to environmental hazards, in the expectation that they would use this information remediate their own risks, did not appear to reduce exposure. This failure may reflect the fact that individuals facing the highest risks of exposure to pollutants also lack the resources to pursue remediation. Economic interventions appeared to be successful only in certain contexts, such as a situation where participants agree on the desirability of a particular outcome. Even with a population of engaged volunteers, continuing incentives may be required to maintain participation. In these populations, providing measures of exposure and of outcomes achieved may help participants identify better strategies to reduce exposures.

In addition to identifying whether interventions were successes or failures, realist review also tries to identify patterns in the included studies that suggest ways to develop other successful interventions. Although there is limited evidence, this review suggests two potential interventions that could be successful in reducing exposure to environmental hazards that merit further study. First, setting standards based on measurable outcomes and allowing state or regional authorities to develop their own strategies to meet those standards may reduce exposure to environmental hazards. Although few studies provided explicit information about the nature of the interventions that they assessed, some implied that this strategy was used in creating interventions that were found to be successful. Second, stricter enforcement of existing laws may improve health outcomes and reduce exposure to environmental hazards, in part by encouraging more exhaustive remediation strategies. Two studies of lead paint remediation assessed existing laws, which can be more strictly enforced in some areas than in others. Developing strategies to improve enforcement of existing laws may be more politically feasible than establishing new restrictions on existing products, and in some cases it has reduce exposures. Improving enforcement of existing laws could be pursued from the regulatory side or through legal action.

These potential interventions are based on weak evidence. Reviewing the studies of interventions showed that there is a need to improve the documentation of the nature of interventions and the evaluation of existing policies. The results demonstrate that there is a wide mix of research designs used to assess interventions and that few of them provide the type of data needed to identify changes, such as baseline data or results from a control group. These difficulties make comparing and combining studies difficult, yet both are necessary to draw lessons about which interventions can be transferred to different environments. In addition, few studies explicitly formulated a testable assumption, which is a requirement to test and develop theories of change.

### Limitations

This review has a number of limitations. First, by design, the range of potential policy interventions explicitly excluded possible organizational interventions that could affect population subgroups (e.g., workplace interventions) as it addressed only population-level interventions. Second, published articles often provided little detail regarding the study methodology or the policy intervention, which made data analysis challenging. The lack of details regarding both the context and mechanisms of policy interventions in studies is a limitation characteristic to the process of realistic review, which has been discussed in previous research [41]. Finally, our decisions with respect to search strategy excluded some articles. Some published research solely addressed prenatal exposure [42], and at least one paper that could have been included was published after the search was completed [43]. We were unable to assess the effects of the exclusion of gray literature.

## Conclusions

Our analysis found that due to the mix of research designs, the different levels of evaluation, the different interventions assessed with the same outcome data, and the different indicators used to assess exposure, that there was little attention paid to context in conducting research. Despite this, authors sometimes reported that context was influential in determining whether interventions were successful.

The focus on mechanisms, or the response to interventions, is promoted in realist enquiry but rarely addressed in evaluations of interventions or research on outcomes. Most evaluations focus on assessing the type of intervention, but different types of interventions can trigger similar mechanisms. The reactions of the population targeted by interventions generate the outcomes that can be studied and compared. As a result, it is important to assess mechanisms in outcomes research. Identifying patterns in mechanisms may generate new interventions that can address environmental hazards that are currently difficult to measure and address.

Overall, this review suggests that regulatory interventions targeted to specific point sources of pollutants have the most support in the existing literature on reducing exposure to environmental hazards. Banning pollutants in situations where the outcomes can be directly measured, either through environmental or human exposure data, are the strategy for which the most evidence for reducing exposure has been generated. These studies also suggest that consistent monitoring of exposure was critical to assessing the success of interventions. However, to assess how interventions could be transferred to other settings, more information is needed about the specific nature of interventions and the context in which they are applied.

The value of a realist review is that it can contextualize existing evidence and identify the mechanisms by which interventions work. However a realist review cannot overcome limitations in the primary data. This review focused only on published studies that provided measurable outcomes dealing with environmental or human exposures. However a broader analysis that included descriptive studies and/or a review of gray literature would be likely to provide further data on areas how policy interventions intended to reduce environmental exposures are implemented, although those study designs would be even more difficult to generalize. In addition, individual interviews with study authors might provide additional information on missing data and specific policy details, however, this was beyond the scope of this review as such findings could not be systematically assessed.

Taken together, the weaknesses of existing research designs assessing exposure to environmental hazards, the limited data available that measure levels of pollutants over time and in different contexts, and the habit of considering multiple interventions simultaneously in research on environmental hazards, suggest that studies evaluating environmental hazards would be more useful to researchers and policymakers if they modified their reporting. At a minimum, the nature of the intervention(s) being assessed could be documented in each study. Adding information on contextual factors that influence implementation would also assist with assessment. These modifications in reporting would allow future reviews to better assess interventions to address environmental hazards.

## Appendix 1

**Table 2:** Data extraction tool and example of data extracted for a regulatory study of truck emissions [16]

Category of intervention	Regulatory
Regulatory policy	airborne pollutants
Year	2011
Author	Dallmann, T.
Title	Effects of Diesel Particle Filter Retrofits and Accelerated Fleet Turnover on Drayage Truck Emissions at the Port of Oakland
Journal	Environmental Science & Technology
Location	Oakland, CA
Study design	Interrupted time series
Study aim	Measure emissions for drayage trucks operating at the Port of Oakland before and after the implementation of diesel particle filter retrofits and truck replacements.
Inclusion criteria	Diesel trucks driving to the Port of Oakland on 7th Street
Exclusion criteria	Trucks entering from other points
Population	3550 trucks passing the field sampling site on four dates in 2009–2010 during sampling periods
Sample size (treatment/control)	3550 trucks, ~70 % estimated to be drayage trucks
Exposure	Air sampling line extended above vertical exhaust stacks of trucks driving below overpass sampling site
Intervention	Diesel particlate filter retrofits and accelerated truck replacement (no breakdown)
Duration of intervention	4 weekday sampling periods ranging from 2.75 to 6.5 h over 7 months (Nov 2009-Jun 2010)
Comparison	Pre- and post-implementation of the California Air Resources Board drayage truck emission control regulation (2010)
Outcomes measures	levels of carbon dioxide, nitrogen dioxide, black carbon, particulate matter
Outcome verification (if self-reported)	air quality samples drawn to gas and particulate phase analyzers
Effect modifiers measured	n/a
Results	CO2: not reported; NO: decrease of 40 %; BC: reduction of 50 %; PM: 5x increase in trucks with no measurable PM, emissions data not reported due to poor instrument response; summary: reductions in BC caused by retrofit/replacement, reductions in NO caused by replacement
Risk of bias	choice of observation dates, concealment of allocation, blinding: not described; incomplete outcome data: emissions for CO2 and PM not reported
Funding source	Bay Area Air Quality Management District, University of California research program in sustainable transportation
Additional information	

## Additional file

Additional file 1: Literature search strategy. (DOCX 28 kb)
