# Supplementary material for: Realist review of policy intervention studies aimed at reducing exposures to environmental hazards in the United States
Source: BMC Public Health. 2016 Aug 18;16:822. doi: 10.1186/s12889-016-3461-7 (PMC4991102; doi:10.1186/s12889-016-3461-7)
Supplement: Additional file 1: — Literature search strategy. (DOCX 28 kb) [file 12889_2016_3461_MOESM1_ESM.docx]

### Additional file 1: Literature search strategy

The final run date for PubMed was May 20, 2014, which produced 5958 references

(Environmental Pollution[majr:noexp] OR Environmental Pollution/analysis[mh:noexp] OR

Environmental Pollution/pc[mh:noexp] OR Air Pollution[majr:noexp] OR Air Pollution/analysis

[mh:noexp] OR Air Pollution/pc[mh:noexp] OR Air Pollution, Indoor[majr] OR Air Pollution,

Indoor/analysis OR Air Pollution, Indoor/pc OR Environmental Exposure[mh:noexp] OR

Environmental Health[majr:noexp] OR Inhalation Exposure/analysis OR Inhalation Exposure/pc

OR Petroleum Pollution/analysis OR Petroleum Pollution/pc OR Waste

Products/analysis[mh:noexp] OR Waste Products/pc[mh:noexp] OR Hazardous Waste[majr] OR

Hazardous Waste/analysis[mh:noexp] OR Hazardous Waste/pc[mh:noexp] OR Industrial

Waste/analysis OR Industrial Waste/pc OR Waste Water/analysis[mh] OR Water

Pollution[majr:noexp] OR Water Pollution/analysis[mh:noexp] OR Water

Pollution/pc[mh:noexp] OR Water Pollution, Chemical[majr:noexp] OR Water Pollution,

Chemical/analysis[mh:noexp] OR Water Pollution, Chemical/pc[mh:noexp] OR Endocrine

Disruptors[majr] OR Endocrine Disruptors/analysis OR Endocrine Disruptors/pc OR

Environmental Pollutants[majr:noexp] OR Environmental Pollutants/analysis[mh:noexp] OR Air

Pollutants/analysis[mh:noexp] OR Soil Pollutants/analysis[mh:noexp] OR Water

Pollutants/analysis[mh:noexp] OR Water Pollutants, Chemical/analysis OR Hazardous

Substances/analysis[mh:noexp] OR Asbestos[majr] OR Beryllium[majr] OR “bisphenol a” OR

Cadmium[majr] OR Cadmium Poisoning[majr] OR Chlorpyrifos[majr] OR Heavy

Metals[majr:noexp] OR Heavy Metals/analysis[mh:noexp] OR Lead[majr] OR Lead

Poisoning[majr] OR Mercury[majr] OR Mercury Poisoning[majr] OR Organophosphates[majr]

OR Organophosphate Poisoning[majr] OR Pesticides[majr] OR Pesticides/analysis OR

Pesticides/pc OR Phthalic Acids[majr] OR Radon[majr] OR Environmental Monitoring[majr]

OR Environment[majr:noexp] OR Atmosphere/analysis[majr:noexp] OR

Air/analysis[majr:noexp] OR Carbon Footprint[majr] OR Fresh Water/analysis[majr] OR

Bays/analysis[majr] OR Soil/analysis[majr] OR Wetlands[majr] OR Wilderness[majr] OR

Conservation of Natural Resources[majr:noexp] OR Energy-Generating Resources[majr] OR

Fossil Fuels/analysis[majr] OR Coal/analysis[majr] OR Gas, Natural/analysis[majr] OR

Petroleum/analysis[majr] OR Vehicle Emissions[majr] OR Vehicle Emissions/analysis OR

Vehicle Emissions/pc OR (biomonitor*[tiab] OR chemical exposure*[tiab] OR hazardous

chemical*[tiab] OR pollut*[tiab] OR toxic substance*[tiab] OR toxics[tiab] AND (analysis[tw]

OR analyze*[tiab] OR assess*[tiab] OR decreas*[tiab] OR measur*[tiab] OR reduce*[tiab] OR

reducing[tiab] OR reduction*[tiab])) ) AND (Policy[majr:noexp] OR Policy Making[majr] OR

Environmental Policy[mh] OR Epidemiological Monitoring[mh] OR Government[majr] OR

Government Regulation [majr] OR Industry[majr:noexp] OR Health Promotion[majr] OR

Interinstitutional Relations[majr] OR Legislation as Topic[majr:noexp] OR “Legislation and

Jurisprudence”[sh] OR Public Health[majr:noexp] OR Social Control, Formal[majr:noexp] OR

Social Control, Informal[majr:noexp] OR Social Control Policies[majr:noexp] OR Health

Policy[majr:noexp] OR Organizational Policy[majr] OR Private Sector[majr] OR Public

Sector[majr] OR Public Policy[majr:noexp] OR Standards[sh] OR Taxes[majr] OR

legislat*[tiab] OR (regulation*[tiab] NOT (cell OR cells OR cellular) ) OR regulatory[tiab] OR

community based strateg*[tiab] OR guideline*[tiab] OR health department*[tiab] OR law[tiab]

OR laws[tiab] OR legislation*[tiab] OR requirements[tiab] OR rule[tiab] OR rules[tiab] OR

ruling*[tiab] OR statute*[tiab] OR penalt*[tiab] OR incentive*[tw] OR tax[tiab] OR taxes[tiab]

OR ban[tiab] OR bans[tiab] OR labeling[tiab] OR mandate*[tiab] OR policies[tiab] OR

policy[tiab] OR "Environmental Protection Agency"[tiab] OR “environmental impact”[tiab] OR

“health impact”[tiab] ) AND (Comparative Study[pt] OR Epidemiologic Studies[mh] OR

Evaluation Studies[pt] OR Intervention Studies[mh] OR Program Evaluation[mh] OR

action[tiab] OR actions[tiab] OR effective*[tiab] OR evaluat*[tiab] OR intervention*[tiab] OR

outcome[tiab] OR outcomes[tiab] OR program[tiab] OR programs[tiab] OR (decrease*[tiab] OR

lower[tiab] OR lowers[tiab] OR prevent*[tiab] OR reduce*[tiab] OR reducing[tiab] OR

reduction*[tiab] AND (concentration*[tiab] OR contaminat*[tiab] OR emission*[tiab] OR

“environmental impact”[tiab] OR exposur*[tiab] OR “health impact”[tiab] OR level[tiab] OR

levels[tiab] OR pollut*[tiab])) OR Risk Assessment[majr:noexp] OR Safety[majr:noexp] OR

overview*[ti]) AND (United States[mh] OR United States[tw] OR "USA"[tiab] OR "U.S."[tiab]

OR "U.S.A."[tiab] OR "USA"[ad] OR “United States”[ad] OR “U.S.A.”[ad] OR “U.S.”[ad] OR

Alabama*[tiab] OR Alaska*[tiab] OR Arizona*[tiab] OR Arkansa*[tiab] OR California*[tiab]

OR Colorado*[tiab] OR Connecticut[tiab] OR Delawar*[tiab] OR Florid*[tiab] OR

Georgia*[tiab] OR Hawaii[tiab] OR Hawai*[tiab] OR Idaho*[tiab] OR Illinois*[tiab] OR

Indiana*[tiab] OR Iowa*[tiab] OR Kansas[tiab] OR Kansan*[tiab] OR Kentuck*[tiab] OR

Louisiana*[tiab] OR Maine[tiab] OR Maryland*[tiab] OR Massachus*[tiab] OR

Michigan*[tiab] OR Minnesota*[tiab] OR Mississippi*[tiab] OR Missour*[tiab] OR

Montana*[tiab] OR Nebraska*[tiab] OR Nevada*[tiab] OR New Hampshir*[tiab] OR New

Jersey*[tiab] OR New Mexico[tiab] OR New Mexican*[tiab] OR New York*[tiab] OR North

Carolina*[tiab] OR North Dakota*[tiab] OR Ohio*[tiab] OR Oklahoma*[tiab] OR

Oregon*[tiab] OR Pennsylvania*[tiab] OR Rhode Island*[tiab] OR South Carolina*[tiab] OR

South Dakota*[tiab] OR Tennessee*[tiab] OR Texas*[tiab] OR Utah*[tiab] OR

Vermont*[tiab] OR Virginia*[tiab] OR Washington*[tiab] OR West Virginia*[tiab] OR

Wiscons*[tiab] OR Wyoming*[tiab) AND English[la] NOT (Animals[mh] NOT Humans[mh])

NOT news[pt] NOT (tobacco OR smok* OR birth* OR prenatal* OR fetus OR fetal OR

pregnan* OR radiation OR occupation* OR “work-related” ) NOT (africa OR americas OR

antarctic regions OR antarctica OR arctic regions OR arctic OR australia OR australian* OR

asia OR china OR europe OR european* OR islands OR oceania NOT united states)

Embase (Elsevier) search strategy

The final run date for Embase was May 20, 2014, which produced 8549 refs

'pollution and pollution related phenomena'/mj OR 'pollution'/mj OR pollutant/exp/mj OR 'air

pollution'/mj OR 'air pollution control'/mj OR 'air monitoring'/mj OR 'ecosystem monitoring'/mj

OR 'water sampling'/mj OR 'enviromental exposure' OR 'environmental health'/mj OR 'waste'/mj

OR 'agricultural waste'/mj OR 'pesticide residue'/mj OR 'hazardous waste'/exp/mj OR 'industrial

waste'/mj OR 'waste water'/mj OR 'waste water management'/mj OR 'water pollution'/mj OR

'water pollution control'/mj OR 'endocrine disruptor'/mj OR 'soil pollution'/mj OR 'soil pollution

control'/mj OR 'spillage'/mj OR 'environmental chemical'/exp/mj OR 'asbestos'/mj OR

'beryllium'/mj OR 'bisphenol a' OR '4,4` isopropylidenediphenol'/mj OR 'cadmium'/mj OR

chloryrifos OR 'heavy metals' OR 'lead'/mj OR 'lead poisoning'/mj OR 'mercury'/mj OR

'mercurialism'/mj OR 'organophosphate poisoning'/mj OR 'phthalic acid'/mj OR 'radon'/mj OR

'environmental monitoring'/mj OR 'environment'/mj OR 'atmosphere'/mj OR 'air'/mj OR 'air

analysis'/mj OR 'carbon footprint'/mj OR 'fresh water'/mj OR 'ground water'/mj OR 'lake

water'/mj OR 'river water'/mj OR 'well water'/mj OR 'aquifer'/mj OR 'bay'/mj OR 'river'/mj OR

'water analysis'/mj OR 'soil'/mj OR 'soil analysis'/mj OR 'wetlands'/mj OR 'wilderness'/mj OR

'biofuel'/mj OR 'fossil fuel'/mj OR 'coal'/mj OR 'natural gas'/mj OR 'petroleum'/mj OR

(biomonitor*:ab,ti OR 'chemical exposure':ab,ti OR 'chemical exposures':ab,ti OR 'hazardous

chemical':ab,ti OR 'hazardous chemicals':ab,ti OR pollut*:ab,ti OR 'toxic substance':ab,ti OR

'toxic substances':ab,ti OR toxics:ab,ti AND (analysis:ab,ti OR analyze*:ab,ti OR assess*:ab,ti

OR decreas*:ab,ti OR measur*:ab,ti OR reduce*:ab,ti OR reducing:ab,ti OR reduction:ab,ti))

AND ('policy'/mj OR 'health care policy'/mj OR 'environmental planning'/mj OR

'epidemiological monitoring'/mj OR 'government'/mj OR 'government regulation'/mj OR

'industry'/mj OR 'health promotion'/mj OR 'law'/mj OR 'public health'/mj OR 'social control'/mj

OR 'reward'/mj OR 'public-private partnership'/mj OR 'standard'/mj OR 'air quality standard'/mj

OR 'tax'/mj OR 'water standard'/mj OR legislat*:ab,ti OR (regulation*:ab,ti NOT (cell OR cells

OR cellular)) OR regulatory:ab,ti OR 'community based strategy':ab,ti OR 'community based

strategies':ab,ti OR guideline*:ab,ti OR 'health department':ab,ti OR 'health departments':ab,ti

OR 'public health service'/mj OR law:ab,ti OR laws:ab,ti OR rule:ab,ti OR rules:ab,ti OR

ruling*:ab,ti OR statute*:ab,ti OR penalt*:ab,ti OR incentive*:ab,ti OR tax:ab,ti OR taxes:ab,ti

OR ban:ab,ti OR bans:ab,ti OR labeling:ab,ti OR mandate*:ab,ti OR policies:ab,ti OR

policy:ab,ti OR 'environmental protection agency':ab,ti OR 'environmental impact assessment'/mj

OR 'health impact assessment'/mj OR 'environmental impact'/mj OR 'environmental impact':ab,ti

OR 'health impact':ab,ti OR 'environmental protection'/de) AND ('united states' OR usa:ab,ti OR

'u.s.':ab,ti OR 'u.s.a.':ab,ti OR usa:ad OR alabama*:ab,ti OR alaska*:ab,ti OR arizona*:ab,ti OR

arkansa*:ab,ti OR california*:ab,ti OR colorado*:ab,ti OR connecticut:ab,ti OR delawar*:ab,ti

OR florid*:ab,ti OR georgia*:ab,ti OR hawaii:ab,ti OR hawai*:ab,ti OR idaho*:ab,ti OR

illinois*:ab,ti OR indiana*:ab,ti OR iowa*:ab,ti OR kansas:ab,ti OR kansan*:ab,ti OR

kentuck*:ab,ti OR louisiana*:ab,ti OR maine:ab,ti OR maryland*:ab,ti OR massachus*:ab,ti OR

michigan*:ab,ti OR minnesota*:ab,ti OR mississippi*:ab,ti OR missour*:ab,ti OR

montana*:ab,ti OR nebraska*:ab,ti OR nevada*:ab,ti OR (new NEXT/1 hampshir*):ab,ti OR

(new NEXT/1 jersey*):ab,ti OR 'new mexico':ab,ti OR (new NEXT/1 mexican*):ab,ti OR (new

NEXT/1 york*):ab,ti OR (north NEXT/1 carolina*):ab,ti OR (north NEXT/1 dakota*):ab,ti OR

ohio*:ab,ti OR oklahoma*:ab,ti OR oregon*:ab,ti OR pennsylvania*:ab,ti OR (rhode NEXT/1

island*):ab,ti OR (south NEXT/1 carolina*):ab,ti OR (south NEXT/1 dakota*):ab,ti OR

tennessee*:ab,ti OR texas*:ab,ti OR utah*:ab,ti OR vermont*:ab,ti OR virginia*:ab,ti OR

washington*:ab,ti OR (west NEXT/1 virginia*):ab,ti OR wiscons*:ab,ti OR wyoming*:ab,ti)

AND ('comparative study'/de OR 'evaluation study'/exp OR 'intervention study'/de OR

action:ab,ti OR actions:ab,ti OR effective*:ab,ti OR evaluation*:ab,ti OR intervention*:ab,ti OR

outcome:ab,ti OR outcomes:ab,ti OR program:ab,ti OR programs:ab,ti OR 'risk assessment'/mj

OR 'safety'/mj OR overview*:ti OR 'clinical study'/de OR 'controlled study'/exp OR 'major

clinical study'/de OR ((decrease* OR lower OR lowers OR prevent* OR reduce* OR reducing

OR reduction*) NEAR/10 (concentration* OR contaminat* OR emission* OR 'environmental

impact' OR exposur* OR 'health impact' OR level OR levels OR pollut*)):ab,ti) AND

[english]/lim NOT ([animals]/lim NOT ([humans]/lim OR 'patient'/exp)) NOT (tobacco OR

smok* OR birth* OR prenatal* OR fetus OR fetal OR pregnan* OR radiation OR occupation*

OR 'work related') NOT (africa OR americas OR antarctic OR antarctica OR arctic OR australia

OR australian* OR asia OR china OR europe OR european* OR islands OR oceania NOT

'united states')

Toxline search strategy

 5/12/14 (375 refs) – Searched “U.S. Environmental policy chemical exposure”

 5/14/14 (5243 results) - United States environmental polic*

 5/14/14 (169 results) - United States environmental polic*, Federal regulation*, health

 5/14/14 (157 results) - United States environmental polic*, policy making, evaluation*,

health, NOT tobacco

 5/14/14 (146 refs) - United States environmental polic*, environmental regulation*,

health, policy evaluation*, NOT tobacco

 5/21/14 (196 results) - (environment OR environmental OR pollution OR pollutant* OR

health OR pesticide*) AND ("policy intervention" OR "policy interventions") NOT

(tobacco OR smok* OR radiation OR alcohol)

Web of Science

A Web of Science cited reference search was conducted on selected references
